# Annotation-efficient deep learning for breast cancer whole-slide image classification using tumour infiltrating lymphocytes and slide-level labels

**DOI:** 10.1038/s44172-024-00246-9

**Published:** 2024-07-25

**Authors:** Rashindrie Perera, Peter Savas, Damith Senanayake, Roberto Salgado, Heikki Joensuu, Sandra O’Toole, Jason Li, Sherene Loi, Saman Halgamuge

**Affiliations:** 1 School of Electrical, Mechanical and Infrastructure Engineering, University of Melbourne, Melbourne, VIC 3010 Australia; 2 Division of Cancer Research, Peter MacCallum Cancer Centre, Melbourne, VIC 3000 Australia; 3 Sir Peter MacCallum Department of Medical Oncology, University of Melbourne, Parkville, VIC 3010 Australia; 4 Department of Pathology, GZA-ZNA Ziekenhuizen, Antwerp, Belgium; 5 Helsinki University Hospital and University of Helsinki, Helsinki, Finland; 6 The Garvan Institute of Medical Research and The Kinghorn Cancer Centre, Sydney, NSW 2010 Australia

**Keywords:** Image processing, Cancer

## Abstract

Tumour-Infiltrating Lymphocytes (TILs) are pivotal in the immune response against cancer cells. Existing deep learning methods for TIL analysis in whole-slide images (WSIs) demand extensive patch-level annotations, often requiring labour-intensive specialist input. To address this, we propose a framework named **an**notation-efficient **s**egmentation and **a**ttention-based **c**lassifier (ANSAC). ANSAC requires only slide-level labels to classify WSIs as having high vs. low TIL scores, with the binary classes divided by an expert-defined threshold. ANSAC automatically segments tumour and stroma regions relevant to TIL assessment, eliminating extensive manual annotations. Furthermore, it uses an attention model to generate a map that highlights the most pertinent regions for classification. Evaluating ANSAC on four breast cancer datasets, we demonstrate substantial improvements over three baseline methods in identifying TIL-relevant regions, with up to 8% classification improvement on a held-out test dataset. Additionally, we propose a pre-processing modification to a well-known method, enhancing its performance up to 6%.

## Abstract (web summary)

Perera et al. developed a machine-learning approach for classifying whole-slide images into binary categories of tumour-infiltrating lymphocytes. They have benchmarked it against two established models and made the entire processing pipeline available as open source.

## Introduction

Tumor-Infiltrating Lymphocytes (TILs) are a type of immune cells that can infiltrate the microenvironment of a tumor. TILs have been recognized as an important biomarker in several types of cancers, including breast cancer, due to their favorable correlations with treatment responses and patient survival^1–4^. TIL assessment typically consists of several steps including but not limited to the identification of regions of interest (ROIs) such as tumor/stroma regions within the tissue section, and the identification of TIL nuclei within the ROIs^5,6^. Although there are best practices in place for TIL assessment^5,6^, the manual assessment of TILs on large tissue sections is a laborious and time-consuming task prone to inter-observer variability and ambiguity^6–8^. Therefore, there is growing interest in using Deep Neural Networks (DNNs) for TIL analysis in whole-slide images (WSIs)^9,10^.

Existing DNNs for TIL analysis divide the WSI, typically of giga-pixel magnitude, into relatively small patches of several hundreds of pixels in width and height and obtain extensive patch-level human annotations^9,11–14^. This tedious and expensive manual task requires domain expertise and is typically performed by pathologists^15,16^. Moreover, the division of WSIs also partially destroys the spatial arrangement and contextual information available in a full WSI required for TIL analysis. In contrast, our goal is to minimize manual supervision by requiring only slide-level annotations while also utilizing the spatial and contextual information present in the WSIs for TIL analysis.

Some existing methods that rely solely on slide-level labels use a pre-trained feature extractor to compress the information within the WSIs. These methods extract a set of feature embeddings from the WSIs before learning a downstream task, such as predicting the presence of metastasis or cancer sub-typing^15,17–21^. There are two different strategies used in these methods. In both strategies, feature embeddings of the patches across the WSI are extracted^15–19^. The first strategy considers the spatial arrangement of patches: it keeps track of the original patch coordinate information and uses the information to learn the downstream task^18,19^. The second strategy does not consider the spatial arrangement: it treats the patches as a permutable set of patches and uses only the features extracted for each patch while learning the downstream task^15,17^. However, to the best of our knowledge, there has been no prior work on using DNNs exclusively based on slide-level TIL labels. Taking these into consideration, we make three contributions:

1. We first adapt and apply two well-known methods that follow the above two strategies to our problem of TIL analysis.
2. We then propose a modification to one of these methods and show the performance improvement.
3. Finally, we highlight the limitations of both strategies for TIL analysis and propose a strategy and a pipeline that addresses these limitations, which is our main contribution.

The first method we investigate is Neural Image Compression (NIC) and its associated convolutional neural network (CNN)^18,19,22^. NIC involves encoding each patch within a WSI using a pre-trained feature extractor and arranging them into a 3D grid while retaining their original patch coordinates. The generated 3D grids along with their corresponding slide-level labels are then used to train a CNN for either classification or regression. Here, NIC only performs a compression of the gigapixel WSIs while the CNN attempts to learn the downstream task using the compressed WSI representations. We designed a CNN architecture, named ${C}_{\theta }$ to handle the sizes of WSIs in our datasets and the combined pipeline of NIC and CNN (${C}_{\theta }$) is referred to as NIC-CNN. NIC is capable of managing gigapixel-sized WSIs while maintaining the spatial and contextual information and has shown promising results on several tasks, including predicting the presence of metastasis and tumor proliferation speeds^18,19,22^. Although NIC accounts for the spatial arrangement of patches, NIC-CNN fails to report similar good performance in TIL-based classification. Manual TIL evaluation requires the identification of ROIs (i.e., tumor/stroma regions) before identifying TIL cells. However, we believe NIC-CNN struggles to perform well in TIL classification as it does not have sufficient capability to infer and locate ROIs only using the slide-level TIL labels of a small number of training examples.

The second method we investigate is clustering-constrained-attention multiple-instance learning (CLAM), which has an inherent disadvantage as it does not consider the spatial arrangement of patches and ignores the patch coordinate information^17^. CLAM is based on the multiple-instance learning (MIL) algorithm^15,17^ where the patches of a WSI are treated as a permutable set of patches. In the conventional MIL algorithm, a WSI is labeled as positive if at least one patch is positive or negative if all patches are negative^17^. CLAM extends MIL for multi-class classification using concepts such as clustering and attention where it uses features extracted from patches as a prior for learning the attention scores. It has reported high performance in the subtyping of renal-cell-carcinoma and non-small-cell-lung-cancer as well as in the detection of lymph node metastasis^17^. Despite these claims, the MIL-based approach in CLAM may not be efficiently applied to TIL analysis in WSIs due to three main limitations. Firstly, similar to NIC-CNN, CLAM does not identify the tumor/stroma regions before identifying TIL regions. Secondly, unlike in NIC-CNN, CLAM does not account for the spatial arrangement of the patches. Thirdly, the feature extractor used in CLAM has been pre-trained on an unrelated natural image dataset (ImageNet^23^), which may lower the ability to extract histologically relevant information from patches^24^.

We propose a way to address CLAM’s third limitation by enhancing its feature extractor through pre-training on histology images, which is our second contribution. This modification involves using momentum-based contrastive learning (MoCo)^25^, a form of self-supervised learning (SSL) to train a feature extractor ${E}_{\theta }$, on histology images instead of natural images. By using histology images for MoCo training, we aim to capture histologically relevant information more effectively^24,26^. We modify the original CLAM architecture to incorporate this change, resulting in the “CLAM-MoCo” architecture. However, the first and second limitations of CLAM remain unchanged.

**Fig. 1:**
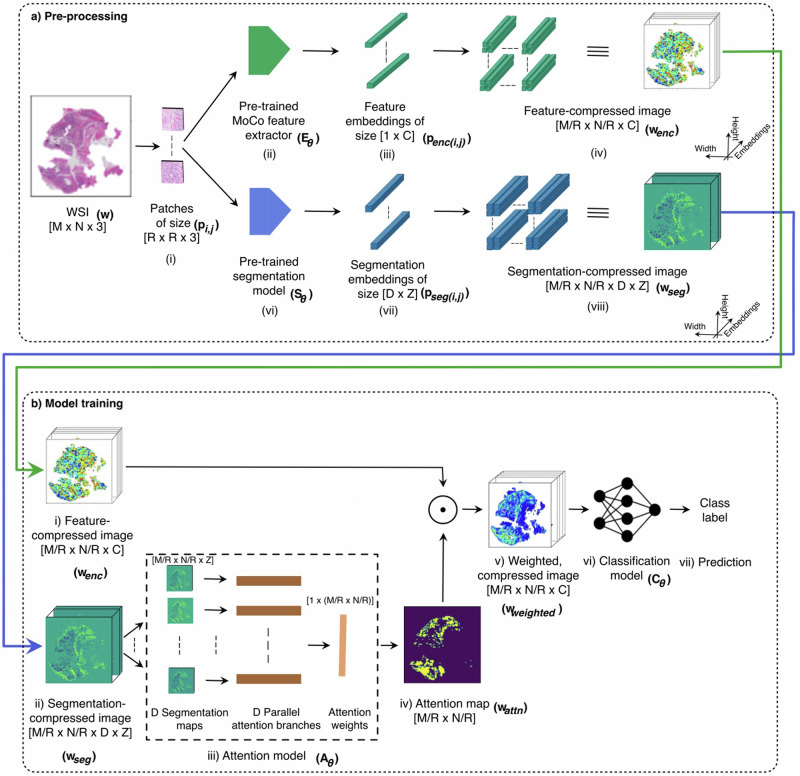
Overview of the ANSAC pipeline. **a** Pre-processing: The WSI, $w$ of height $M$ and width $N$ is divided into patches ${p}_{i,j}$ of height and width $R$ and mapped to low-dimensional feature embedding vectors, ${p}_{{enc}(i,j)}$ of size $1\times C$ using a pre-trained feature-extractor, ${E}_{\theta }$ (top branch) and low-dimensional segmentation embedding vectors, ${p}_{{seg}(i,j)}$ of size $D\times Z$ using a pre-trained segmentation model, ${S}_{\theta }$ (bottom branch). The extracted ${p}_{{enc}(i,j)}$ and ${p}_{{seg}(i,j)}$ vectors are placed into two 3D grids preserving their original coordinates, resulting in a feature-compressed representation, ${w}_{{enc}}$ of size $M/R\times N/R\times C$ and a segmentation compressed representation, ${w}_{{seg}}$ of size $M/R\times N/R\times D\times Z$, respectively. **b** Model training: ${w}_{{seg}}$ is used to train an attention model, ${A}_{\theta }$ to generate an attention map, ${w}_{{attn}}$. ${w}_{{attn}}$ is then combined with ${w}_{{enc}}$ from (a-iv) to obtain a weighted, compressed feature representation for the WSI,${w}_{{weighted}}$ which is processed through a classification network ${C}_{\theta }$ to predict a binary class label. The default values used in this study are $M=N=20480,R=256,C=128,D=6$ and $Z=576$ where $M$ and $N$ are the size of the gigapixel image, $R$ is the size of the square patches$,C$ is the size of feature embedding vectors,$D$ is the number of regions identified by the segmentation model, and $Z$ is the size of segmentation embedding vectors.

We propose the annotation-efficient **s**egmentation and **a**ttention-based **c**lassifier (ANSAC) framework illustrated in Fig. 1, which overcomes the limitations in the above methods as described below:

1. Unlike NIC-CNN, CLAM and CLAM-MoCo, ANSAC employs an auxiliary, pre-trained segmentation module^13^ (Fig. 1a-vi) to identify ROIs in the WSI, without requiring explicit manual marking or annotation of the ROIs.
2. Unlike NIC-CNN, ANSAC uses an attention model to identify the specific areas of high importance within the ROIs for the classification task.
3. Unlike CLAM and CLAM-MoCo, ANSAC accounts for the contiguity of patches mapped into ROIs where TILs are scored.
4. Unlike CLAM, ANSAC uses a histologically relevant MoCo-based feature (Fig. 1a-ii) extractor to extract features from WSI patches.

Importantly, in comparison to existing computational methods that specifically focus on TIL analysis, ANSAC comprises of several unique contributions. Firstly, ANSAC represents a notable advancement in terms of annotation efficiency. Unlike existing TIL methods that necessitate manual annotations of TIL nuclei or regions at the patch level^9,11,12,27^, ANSAC streamlines the annotation process by requiring manual annotation only at the slide-level. This distinction greatly reduces the need for patch-level annotations by the users, making the training process more scalable for large datasets. Secondly, ANSAC is the first computational TIL algorithm that introduces the utilization of attention-based mechanisms for distinguishing TIL-relevant ROIs. This innovative approach allows the model to focus on specific areas within the slide that are most indicative of TIL presence, enhancing the accuracy and interpretability of the analysis. Finally, in contrast to existing methods that ignore spatial information within the WSI^9,11^, ANSAC explicitly accounts for the spatial information between patches when predicting the final slide-label. Overall, ANSAC stands out as a computational method in the field of TIL analysis, characterized by its annotation efficiency, utilization of attention-based mechanisms, and integration of spatial information.

One of the key features of ANSAC is its segmentation module. This module helps ANSAC to become annotation-efficient by only requiring manual input at the slide-level to learn the classification task. The segmentation module has been trained to identify $D=6$ number of ROIs, namely tumor, stroma, lymphocyte infiltrates, necrosis, other (rare) regions and background region^13^. ANSAC uses an intermediate layer from the module to produce a segmentation prediction for an input image. This prediction consists of $D$ maps, with each map corresponding to a specific ROI identified by the segmentation module. Unlike CLAM which uses patch features for learning attention, ANSAC uses the output from the segmentation module as a prior for learning attention. As shown in the reported results, ANSAC’s ability to use segmentation-based priors is beneficial in tasks such as TIL-based classification where identification of specific regions (i.e., tumor/stroma regions) should be done before determining the final prediction.

The pre-processing stage in ANSAC (shown in Fig. 1a) first divides the WSI $w$ into patches ${p}_{i,j}$ (Fig. 1a-i) and processes the patches through a pre-trained feature extractor ${E}_{\theta }$ (Fig. 1a-ii), resulting in low-dimensional feature embeddings denoted as ${p}_{{enc}(i,j)}$ (Fig. 1a-iii). These embeddings are then arranged into a multi-dimensional array that preserves the original spatial layout of the patches resulting in a feature-compressed representation of the WSI named as ${w}_{{enc}}$ (Fig. 1a-iv). To obtain histologically rich feature embeddings, we use the same feature extractor ${E}_{\theta }$ that we introduced for CLAM-MoCo as ANSAC’s feature extractor. Similar to the feature compression, ANSAC also compresses the WSI by processing the patches in an annotation-efficient manner through the pre-trained segmentation model^13^ ${S}_{\theta }$ (Fig. 1a-vi → a-vii → a-viii) to obtain a segmentation-compressed representation of the WSI denoted by ${w}_{{seg}}$.

The model training stage in ANSAC (shown in Fig. 1b) uses the feature and segmentation compressed representations of the WSI, i.e., ${w}_{{enc}}$ (Fig. 1b-i) and ${w}_{{seg}}$ (Fig. 1b-ii) to learn the classification task. ${w}_{{seg}}$ is split into $D$ separate region-specific maps and input into the attention model ${A}_{\theta }$ (Fig. 1b-iii) which comprises of $D$ parallel branches, one for each ROI identified by the segmentation model. The weights from the $D$ branches are summed up and normalized for each patch, resulting in a final attention weight. The attention weight of each patch is then arranged into a 2-dimensional grid in the corresponding original patch coordinates to obtain the attention map named as ${w}_{{attn}}$ (Fig. 1b-iv). This map is combined with ${w}_{{enc}}$, creating a weighted, compressed WSI representation denoted by ${w}_{{weighted}}$ (Fig. 1b-v). Finally, ${w}_{{weighted}}$ is input into a CNN (Fig. 1b-vi), which predicts a binary class label. For a fair comparison, we use the same CNN architecture ${C}_{\theta }$ that was used in the NIC-CNN method.

We evaluate the performance of ANSAC in comparison to NIC-CNN, CLAM, and CLAM-MoCo for the classification of high vs. low TIL scores in WSIs using four breast cancer WSI datasets: CLEOPATRA^28^ $({D}_{{cleo}})$, FinHER^29^ $({D}_{{fin}})$, The Cancer Genome Atlas Breast Invasive Carcinoma cohort (TCGA-BRCA) $\left({D}_{{tcga}}\right)$ and TIGER (${D}_{{tiger}}$) where ${D}_{{cleo}}$ and ${D}_{{fin}}$ are in-house datasets while ${D}_{{tcga}}$ and ${D}_{{tiger}}$ are publicly available datasets. We use ${D}_{{cleo}}$, ${D}_{{fin}}$ and ${D}_{{tcga}}$ datasets for model training, validation, and testing while ${D}_{{tiger}}$, is used as an independent test only dataset. It is worth noting that the NIC-CNN pipeline is made up of the following steps in Fig. 1bi → vi → vii. We observe that ANSAC has superior performance over NIC-CNN, CLAM, CLAM-MoCo across most datasets and that CLAM-MoCo is the second best. Moreover, in cases where segmentation labels are unavailable, CLAM-MoCo may offer a viable alternative to obtain inference with comparable performance to ANSAC.

## Results

### Datasets

Tissue images are commonly preserved for further analysis using either Formalin Fixation Paraffin Embedding (FFPE) or Fresh Freezing (FF). FFPE is a preservation method that enables long-term storage and accurate interpretation of tissue morphologies, while FF is typically prone to introducing misleading histological artefacts that can hinder accurate interpretation by pathologists^30^. However, FF slides are still popularly used for clinical decision making due to its fast acquisition time (~15 min) compared to that of FFPE’s (~36 h)^30^. Datasets ${D}_{{cleo}}$, ${D}_{{fin}}$, and ${D}_{{tiger}}$ are comprised of FFPE-preserved tissues, while ${D}_{{tcga}}$ contains FF tissues (refer Supplementary Table 1 for more information). To evaluate the model’s performance on mixtures of tissues, we also consider two different mixtures of the individual datasets: ${D}_{{mix\_FFPE}}$, which contains a mixture of only FFPE tissues and ${D}_{{mix\_all}}$, which contains a mixture of both FFPE and FF tissues (refer Table 1). Each individual and combined dataset is made of a training set for model training (${D}_{i}^{{train}}$), validation set for model selection (${D}_{i}^{{valid}}$), and testing set for evaluation (${D}_{i}^{{test}}$). We first divided ${D}_{{cleo}},$ ${D}_{{fin}}$, ${and}$ ${D}_{{tcga}}$ datasets into the three splits as training, validation, and testing following the $60-20-20$ proportion (refer Supplementary Table 2 for more information). The two combined dataset’s training, validation, and testing splits were then formed by concatenating the corresponding splits of the individual datasets.

**Table 1:** Number of WSIs present in the individual (2nd–4th columns), combined (5th-6th columns) and independent test (7th column) datasets where ${D}_{{cleo}}$, ${D}_{{fin}},{D}_{{tcga}}$, and ${D}_{{tiger}}$ correspond to the CLEOPATRA^28^, FinHER^29^, The Cancer Genome Atlas Breast Invasive Carcinoma cohort (TCGA-BRCA) and TIGER datasets while ${D}_{{mix\_FFPE}}$ and ${D}_{{mix\_all}}$ correspond to the combined datasets

Dataset	${D}_{{cleo}}$	${D}_{{fin}}$	${D}_{{tcga}}$	${D}_{{mix\_FFPE}}({D}_{{cleo}}+{D}_{{fin}})$	${D}_{{mix\_all}}({D}_{{cleo}}+{D}_{{fin}}+{D}_{{tcga}})$	${D}_{{tiger}}$(Test only)
Tissue Type	FFPE	FFPE	FF	FFPE	FFPE + FF	FFPE
Train	477	416	444	893	1337	–
Validation	120	104	112	224	336	–
Test	150	131	139	281	420	82

We report classification performance in the format of mean ± standard deviation across ten runs using different weight initializations and the Area under the Receiver Operating Characteristic Curve (AUC) metric^9,10^, while additional metrics such as Sensitivity, Specificity and Accuracy are reported in Supplementary Tables 3–12. Lastly, we generate heatmaps from an intermediate layer of the trained models to visually compare their ability to identify TIL-relevant regions, such as tumor and stroma regions, during the classification task.

### Comparison of ANSAC against other methods

The results reported by the four evaluated methods on the five individual and combined test datasets (2nd–6th columns in Table 1) are reported in Table 2. We trained one model each for all four methods using the corresponding training dataset $({D}_{i}^{{train}})$, validated with the corresponding validation dataset $({D}_{i}^{{valid}})$ and evaluated it on the corresponding testing dataset $({D}_{i}^{{test}})$.

**Table 2:** AUC scores reported for each model trained on ${D}_{i}^{{train}}$ and evaluated on ${D}_{i}^{{test}}$, where $i={cleo},{fin},{tcga},{mix\_FFPE},{and}$ ${mix\_all}$

Method	${D}_{{cleo}}^{{test}}$	${D}_{{fin}}^{{test}}$	${D}_{{tcga}}^{{test}}$	${D}_{{mix\_FFPE}}^{{test}}({D}_{{cleo}}^{{test}}+{D}_{{fin}}^{{test}})$	${D}_{{mix\_all}}^{{test}}({D}_{{cleo}}^{{test}}+{D}_{{fin}}^{{test}}+{D}_{{tcga}}^{{test}})$
NIC-CNN	$89.95 \% \pm 0.013$	$90.72 \% \pm 0.022$	$86.92 \% \pm 0.015$	$87.74 \% \pm 0.011$	$87.45 \% \pm 0.010$
CLAM	$83.51 \% \pm 0.023$	$93.46 \% \pm 0.018$	$79.27 \% \pm 0.078$	$88.65 \% \pm 0.008$	$87.16 \% \pm 0.009$
CLAM-MoCo	$90.05 \% \pm 0.012$	${{{{\boldsymbol{95}}}}}{{{{\boldsymbol{.}}}}}{{{{\boldsymbol{77}}}}} \% {{{{\boldsymbol{\pm }}}}}{{{{\bf{0}}}}}{{{{\boldsymbol{.}}}}}{{{{\bf{010}}}}}$	${{{{\boldsymbol{87}}}}}{{{{\boldsymbol{.}}}}}{{{{\boldsymbol{87}}}}} \% {{{{\boldsymbol{\pm }}}}}{{{{\bf{0}}}}}{{{{\boldsymbol{.}}}}}{{{{\bf{015}}}}}$	$88.96 \% \pm 0.008$	$88.35 \% \pm 0.008$
ANSAC	${{{{\bf{91}}}}}{{{{\boldsymbol{.}}}}}{{{{\bf{84}}}}} \% {{{{\boldsymbol{\pm }}}}}{{{{\bf{0}}}}}{{{{\boldsymbol{.}}}}}{{{{\bf{007}}}}}$	$95.29 \% \pm 0.015$	$85.74 \% \pm 0.025$	${{{{\bf{89}}}}}{{{{\boldsymbol{.}}}}}{{{{\bf{91}}}}} \% {{{{\boldsymbol{\pm }}}}}{{{{\boldsymbol{0}}}}}{{{{\boldsymbol{.}}}}}{{{{\boldsymbol{017}}}}}$	${{{{\bf{89}}}}}{{{{\boldsymbol{.}}}}}{{{{\bf{24}}}}} \% {{{{\boldsymbol{\pm }}}}}{{{{\boldsymbol{0}}}}}{{{{\boldsymbol{.}}}}}{{{{\boldsymbol{008}}}}}$

The best results are highlighted in bold.

${D}_{{cleo}}^{{test}}$, ${D}_{{fin}}^{{test}},{and}$ ${D}_{{tcga}}^{{test}}$ correspond to the CLEOPATRA^28^, FinHER^29^, and The Cancer Genome Atlas Breast Invasive Carcinoma cohort (TCGA-BRCA) test datasets while ${D}_{{mix\_FFPE}}^{{test}}$ and ${D}_{{mix\_all}}^{{test}}$ correspond to the combined test datasets.

We first compare ANSAC with NIC-CNN, where ANSAC can be seen as an extension of NIC-CNN that incorporates segmentation information. The effectiveness of ANSAC’s unique segmentation map enabled attention model is demonstrated in Table 2, where ANSAC outperformed NIC-CNN on most individual and combined datasets except in ${D}_{{tcga}}^{{test}}$. ANSAC’s slightly lower performance the ${D}_{{tcga}}^{{test}}$ dataset possibly demonstrates a limitation of ANSAC’s segmentation module which was trained exclusively on FFPE data whereas ${D}_{{tcga}}^{{test}}$ contains FF tissues only (see Methods).

Next, we compare ANSAC with CLAM, where ANSAC consistently outperformed CLAM models by $8.33 \%$ for ${D}_{{cleo}}^{{test}}$, $1.83 \%$ for ${D}_{{fin}}^{{test}}$, $6.47 \%$ for ${D}_{{tcga}}^{{test}}$, $1.26 \%$ for ${D}_{{mix\_FFPE}}^{{test}}$ and $2.08 \%$ for ${D}_{{mix\_all}}^{{test}}$. This outcome validates our assumption that spatial and segmentation information is essential for the task at hand. We also observe that CLAM-MoCo outperformed CLAM across all test datasets, emphasizing the advantage of using histology-based feature extractors in digital pathology. Further, CLAM-MoCo also consistently outperformed NIC-CNN demonstrating the importance of accounting for attention region identification in classification.

Finally, we compare ANSAC and CLAM-MoCo to validate the importance of adding segmentation information for attention calculation. ANSAC showed clear superior performance over CLAM-MoCo across the two mixed datasets (${D}_{{mix\_FFPE}}^{{test}}$ and ${D}_{{mix\_all}}^{{test}}$). Among the two FFPE datasets, ANSAC had superior performance on ${D}_{{cleo}}^{{test}}$, while having competitive, second-best performance on ${D}_{{fin}}^{{test}}$. However, ANSAC’s performance on the ${D}_{{tcga}}^{{test}}$ dataset was lower compared to that of CLAM-MoCo. This confirms our previous observation that ANSAC’s segmentation module specializing in FFPE data resulted in the lower performance on ${D}_{{tcga}}^{{test}}$. However, the result reported for the FF tissue sections in ${D}_{{tcga}}^{{test}}$ by ANSAC (~85% AUC) is still promising and can be improved with more appropriate training of the segmentation module.

### Performance on an independent test dataset

The trained models from Table 2 are used to obtain classification predictions on an independent test dataset, ${D}_{{tiger}}$. Since models trained on the larger and more diverse datasets have a better opportunity to generalize during their training, we expect the models trained on ${D}_{{mix\_FFPE}}^{{train}}$ and ${D}_{{mix\_all}}^{{train}}$ to perform better on ${D}_{{tiger}}$. As illustrated in the results shown in Table 3, this assumption is verified. ANSAC demonstrated superior performance compared to other methods across both combined datasets, indicating its robustness in handling WSIs from new data sources without the need for fine-tuning. The models trained on the three individual datasets generally underperformed on ${D}_{{tiger}}$, with only two exceptions (ANSAC trained on ${D}_{{cleo}}^{{train}}$ and CLAM-MoCo trained on ${D}_{{fin}}^{{train}}$). This is likely due to their limited generalizability compared to the models trained on the combined datasets. Notably, the two exceptions achieve somewhat good AUCs only due to their skewed predictions, as shown in the metrics reported in Supplementary Tables 8 and 9.

**Table 3:** AUC scores reported for ${D}_{{tiger}}$ from models trained on ${D}_{i}^{{train}}$, where $i={cleo},{fin},{tcga},{mi}{x}_{{FFPE}},{andmi}{x}_{{all}}.{D}_{{cleo}}^{{train}}$, ${D}_{{fin}}^{{train}},{and}$ ${D}_{{tcga}}^{{train}}$ correspond to the CLEOPATRA^28^, FinHER^29^, and The Cancer Genome Atlas Breast Invasive Carcinoma cohort (TCGA-BRCA) train datasets while ${D}_{{mix}{{{{\rm{\_}}}}}{FFPE}}^{{train}}$ and ${D}_{{mix}{{{{\rm{\_}}}}}{all}}^{{train}}$ correspond to the combined train datasets

Method	Training dataset				
	${D}_{{cleo}}^{{train}}$	${D}_{{fin}}^{{train}}$	${D}_{{tcga}}^{{train}}$	${D}_{{mix\_FFPE}}^{{train}}({D}_{{cleo}}^{{train}}+{D}_{{fin}}^{{train}})$	${D}_{{mix\_all}}^{{train}}({D}_{{cleo}}^{{train}}+{D}_{{fin}}^{{train}}+{D}_{{tcga}}^{{train}})$
NIC-CNN	$77.77 \% \pm 0.0249$	$56.92 \% \pm 0.0590$	$66.05 \% \pm 0.0391$	$81.92 \% \pm 0.026$	$81.87 \% \pm 0.026$
CLAM	$71.38 \% \pm 0.0343$	$73.98 \% \pm 0.0486$	$62.03 \% \pm 0.0636$	$76.82 \% \pm 0.021$	$75.44 \% \pm 0.037$
CLAM-MoCo	$78.97 \% \pm 0.0187$	$82.54 \% \pm 0.0316$	$66.03 \% \pm 0.0407$	$81.71 \% \pm 0.016$	$80.98 \% \pm 0.021$
ANSAC	$80.06 \% \pm 0.0352$	$66.45 \% \pm 0.0945$	$65.76 \% \pm 0.0468$	${{{{\bf{85}}}}}{{{{\boldsymbol{.}}}}}{{{{\bf{13}}}}}{{{{\boldsymbol{ \% }}}}}{{{{\boldsymbol{\pm }}}}}{{{{\boldsymbol{0}}}}}{{{{\boldsymbol{.}}}}}{{{{\boldsymbol{02}}}}}{{{{{\boldsymbol{0}}}}}}^{{{{{\boldsymbol{* }}}}}}$	${{{{\bf{83}}}}}{{{{\boldsymbol{.}}}}}{{{{\bf{40}}}}}{{{{\boldsymbol{ \% }}}}}{{{{\boldsymbol{\pm }}}}}{{{{\boldsymbol{0}}}}}{{{{\boldsymbol{.}}}}}{{{{\boldsymbol{02}}}}}{{{{{\boldsymbol{2}}}}}}^{{{{{\boldsymbol{* }}}}}{{{{\boldsymbol{* }}}}}}$

The best results are highlighted in bold.

* and ** denote the top two accuracies, respectively.

### ANSAC performance on FFPE vs FF tissues

While FFPE slides are more frequently used in the studies reported^17–19^, the impact of having both FFPE and FF tissues in model training is an area yet to be explored for TIL-based classification. Therefore, we investigate in Table 4, the robustness of the most promising method, ANSAC trained on ${D}_{{mix\_FFPE}}^{{train}}$ (only including FFPE data), ${D}_{{tcga}}^{{train}}$ (only including FF data) and ${D}_{{mix\_all}}^{{train}}$ (including both FFPE and FF data), considering their corresponding test datasets ${D}_{{mix\_FFPE}}^{{test}}$, ${D}_{{tcga}}^{{test}}$ and ${D}_{{mix\_all}}^{{test}}$ using AUC scores. Additionally, to quantitatively investigate the overall performance of the three models trained on the three training datasets and tested on each of the three testing datasets, we use the rank and average rank metric^31^. The rank summarizes the mean AUC reported for each test dataset by a model, where the model receives rank 1 if it has the highest AUC, rank 2 if it has the second highest, and so on. The average rank is an average of the individual ranks across the three test datasets (where the lower the average rank is, the better the model is).

**Table 4:** AUC metrics of ANSAC models on ${D}_{{mix\_FFPE}}^{{test}}$ (only including FFPE data), ${D}_{{tcga}}^{{test}}$ (only including FF data) and ${D}_{{mix\_all}}^{{test}}$(including both FFPE and FF data) where ${D}_{{mix\_all}}^{{test}}$ = ${D}_{{mix\_FFPE}}^{{test}}+{D}_{{tcga}}^{{test}}$. ${D}_{{tcga}}^{{test}}$ correspond to the Cancer Genome Atlas Breast Invasive Carcinoma cohort (TCGA-BRCA) test dataset while ${D}_{{mix\_FFPE}}^{{test}}$ and ${D}_{{mix\_all}}^{{test}}$ correspond to the combined test datasets

Training dataset	Testing dataset						
	${D}_{{mix\_FFPE}}^{{test}}$(FFPEonly)		${D}_{{tcga}}^{{test}}({FFonly})$		${D}_{{mix\_all}}^{{test}}$(${FFPE}+{FF}$)		Average rank
	AUC	Rank	AUC	Rank	AUC	Rank	
${D}_{{mix\_FFPE}}^{{train}}$(FFPEonly)	$89.91 \% \pm 0.017$	2	$80.34 \% \pm 0.024$	3	$86.74 \% \pm 0.007$	2	2.3
${D}_{{tcga}}^{{train}}({FFonly})$	$71.94 \% \pm 0.0370$	3	$85.74 \% \pm 0.025$	2	$76.76 \% \pm 0.0289$	3	2.7
${D}_{{mix\_all}}^{{train}}$(${FFPE}+{FF}$)	${{{{\bf{90}}}}}{{{{\boldsymbol{.}}}}}{{{{\bf{20}}}}}{{{{\boldsymbol{ \% }}}}}{{{{\boldsymbol{\pm }}}}}{{{{\boldsymbol{0}}}}}{{{{\boldsymbol{.}}}}}{{{{\boldsymbol{008}}}}}$	1	${{{{\bf{86}}}}}{{{{\boldsymbol{.}}}}}{{{{\bf{92}}}}}{{{{\boldsymbol{ \% }}}}}{{{{\boldsymbol{\pm }}}}}{{{{\boldsymbol{0}}}}}{{{{\boldsymbol{.}}}}}{{{{\boldsymbol{017}}}}}$	1	${{{{\bf{89}}}}}{{{{\boldsymbol{.}}}}}{{{{\bf{24}}}}}{{{{\boldsymbol{ \% }}}}}{{{{\boldsymbol{\pm }}}}}{{{{\bf{0}}}}}{{{{\boldsymbol{.}}}}}{{{{\bf{008}}}}}$	1	**1**

The best results are highlighted in bold.

As expected, the model trained on ${D}_{{mix\_FFPE}}^{{train}}$ performs relatively better on ${D}_{{mix\_FFPE}}^{{test}}$ than on ${D}_{{tcga}}^{{test}}$ as its training only specialises on FFPE slides. Similarly, the model trained on ${D}_{{tcga}}^{{train}}$ showed relatively better performance on ${D}_{{tcga}}^{{test}}$ compared to ${D}_{{mix\_FFPE}}^{{test}}$ since it was trained only on FF slides. However, both models exhibit lower performance when dealing with tissue types that were not encountered during their respecting training. In contrast to these, the model trained on ${D}_{{mix\_all}}^{{train}}$ (which has a larger training dataset including both FFPE and FF slides) consistently performed the best on ${D}_{{mix\_FFPE}}^{{test}}$, ${D}_{{tcga}}^{{test}}$ and ${D}_{{mix\_all}}^{{test}}$. As shown in Table 4, the model trained on ${D}_{{mix\_all}}^{{train}}$ reports the best average rank while models trained on ${D}_{{mix\_FFPE}}^{{train}}$ and ${D}_{{tcga}}^{{train}}$ report the second and third best average rank, respectively. While it was anticipated that the model trained on ${D}_{{mix\_all}}^{{train}}$ would performance well on ${D}_{{mix\_all}}^{{test}}$, it is noteworthy that it outperformed both the models trained on ${D}_{{mix\_FFPE}}^{{train}}$ and ${D}_{{tcga}}^{{train}}$ when evaluated on their respective test datasets. This finding emphasizes the effectiveness of incorporating both FFPE and FF tissues for more robust and generalized model training.

### Interpretability and whole-slide attention visualization

The lack of interpretability and explainability of DNNs can lead to mistrust and hinder their adoption in real-world settings^10,32,33^. Therefore, recent work attempt to visualize and interpret the output of intermediate layers of DNNs to provide clear explanations of model predictions^17,34^. One such approach is to use heatmaps or class activation maps, which are constructed by overlaying an intermediate output from the DNN over the original image^17,34^. These heatmaps highlight regions in the image that are highly activated and thus contribute more towards the class predictions.

**Fig. 2:**
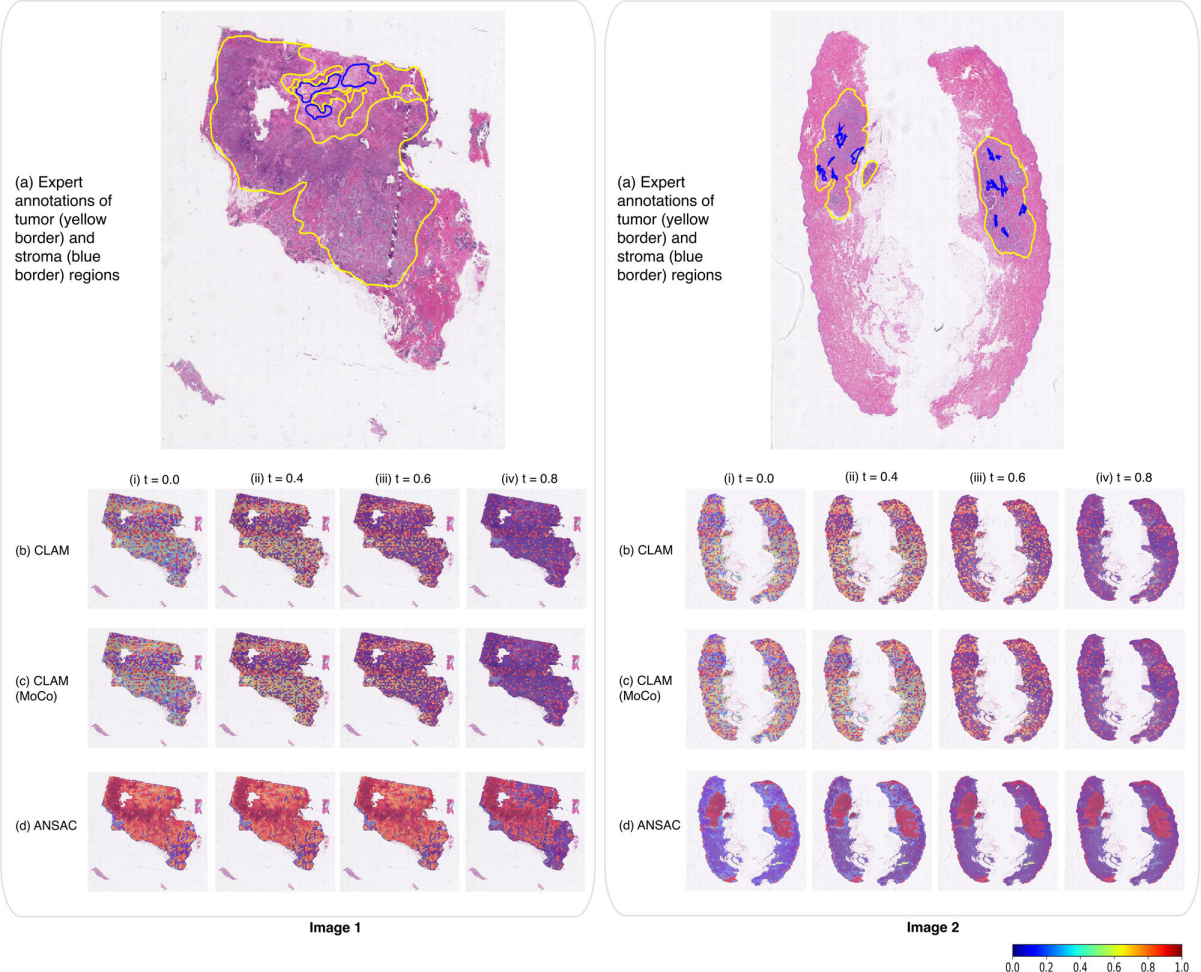
Visualization and interpretability of model predictions of low TIL infiltrated WSIs. Two example slide images with low TIL infiltration are presented in the left and right columns. Here we compare (**a**) the manual annotations of tumor (yellow line) and stroma (blue line) regions with the model predictions from (**b**) CLAM (**c**) CLAM (MoCo) and (**d**) ANSAC for four different threshold settings, $t$ (where $t=\left(i\right)\,0,\left({ii}\right)\,0.4,\left({iii}\right)\, 0.6{and}\left({iv}\right)\, 0.8$). In the heatmaps, red regions have the highest attention, green regions have medium attention and blue regions have the lowest attention values (a color bar is provided at the bottom).

**Fig. 3:**
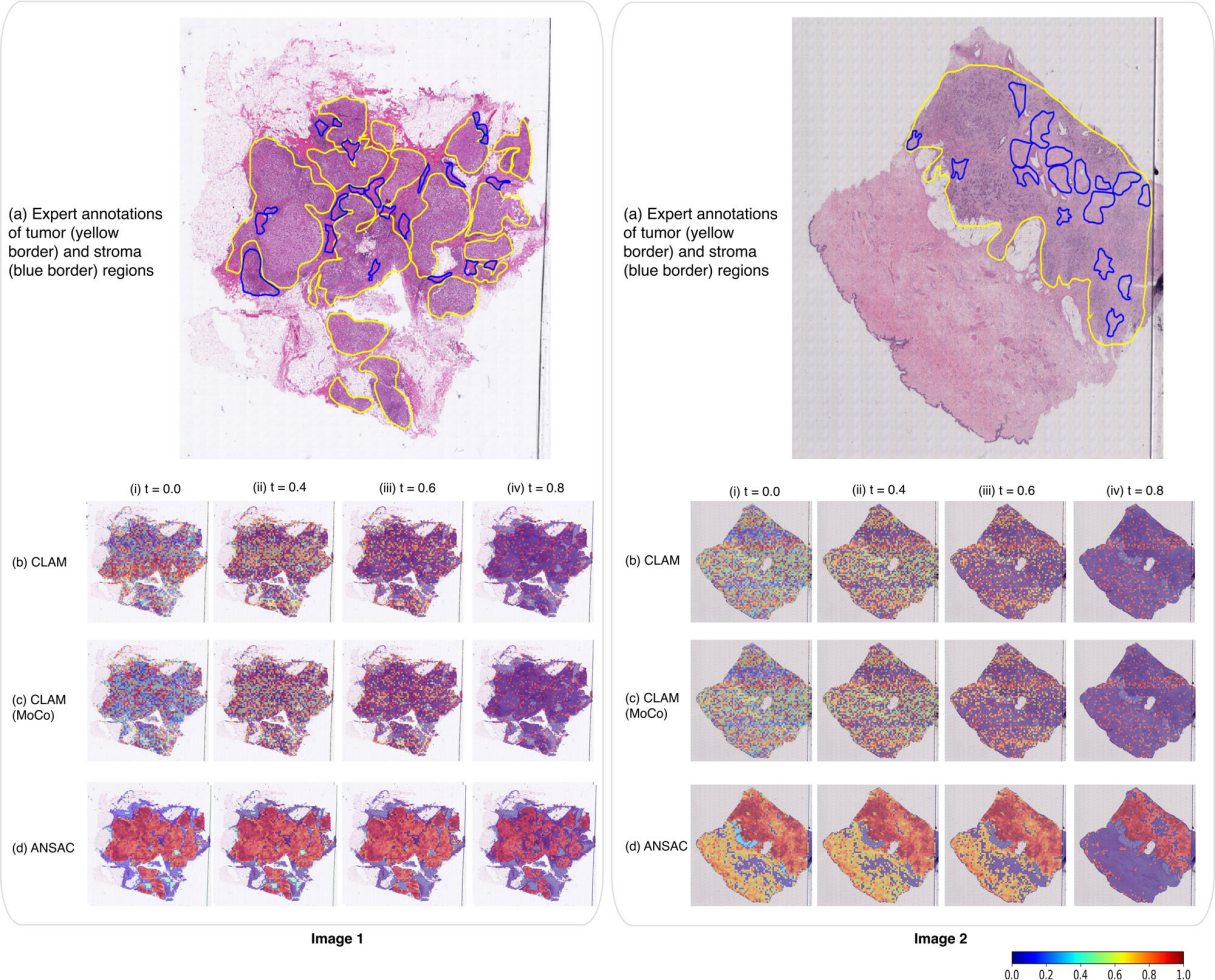
Visualization and interpretability of model predictions of high TIL infiltrated WSIs. Two example slide images with high TIL infiltration are presented in the left and right columns. Here we compare (**a**) the manual annotations of tumor (yellow line) and stroma (blue line) regions with the model predictions from (**b**) CLAM (**c**) CLAM (MoCo) and (**d**) ANSAC for four different threshold settings, $t$ (where $t=\left(i\right)\, 0,\left({ii}\right)\, 0.4,\left({iii}\right)\, 0.6{and}\left({iv}\right)\, 0.8$). In the heatmaps, red regions have the highest attention, green regions have medium attention and blue regions have the lowest attention values (a color bar is provided at the bottom).

Each of the three attention-based methods, CLAM, CLAM-MoCo, and ANSAC consists of an attention model within their pipeline. To visualize and verify the output of the attention models, we generated heatmaps for the attention values predicted by the three models trained on our largest training dataset, ${D}_{{mix\_all}}^{{train}}$ and compared them against manual ROI annotations^17^. Figure 2 shows the heatmaps of two WSIs with low TIL infiltration, while Fig. 3 shows the heatmaps of two WSIs with high TIL infiltration. In both Figs. 2 and 3, the top row illustrates the original WSI with manual ROI annotations provided by an expert breast pathologist, while the next three rows depict the heatmaps generated by CLAM, CLAM-MoCo and ANSAC, respectively. The original WSI is annotated with tumor (yellow line) and stroma (blue line) regions while the generated heatmaps are visualized under four threshold settings, $t$, ${{{{\rm{where}}}}}$ $t={{{\mathrm{0,0.4,0.6}}}}$ ${and}$ $0.8$. For each threshold $t$, we filter out attention values < $t$ such that only attention values greater than $t$ are retained for heatmap generation. Due to limited space, we have moved the heatmaps generated for $t=0.2$ to the Supplementary Figs. 1 and 2.

The heatmaps generated at $t=0$ (i.e., without any filtering) show that the heatmaps generated from CLAM and CLAM-MoCo contain an inconsistent mixture of attention values without any clear patterns or region separations, making it challenging to draw meaningful conclusions from them. In contrast, the heatmaps generated from ANSAC show a clear separation between regions with high and low attention values. Notably, upon closer examination of the heatmaps, we observe that the regions marked with high attention values (colored in dark red color) by ANSAC align closely with the manual ROI annotations. This observation is further validated as we vary $t$ from $0.4$ to $0.8$. With increasing $t$, the heatmaps gradually filter out low attention values, enhancing the visualization of regions with higher attention scores. Here, we observe that both CLAM and CLAM-MoCo predominantly award very low attention values to most tissue regions. Moreover, the heatmaps generated at $t=0.8$ demonstrate that the high attention regions predicted by these models are distributed throughout the image without clear localization. In comparison, ANSAC consistently assigns higher attention values to regions corresponding to manual ROI annotations, as evident in the heatmaps generated at $t=0.8$. Overall, the visual similarities observed between the manual annotations and heatmaps generated by ANSAC provide further evidence of ANSAC’s effectiveness in learning classification tasks in an annotation-efficient manner.

## Discussion

In this paper, our primary aim is to advance computational methods for the highly challenging task of TIL-based WSI classification. We introduce ANSAC, a WSI classification pipeline that performs a binary classification of high versus low levels of TIL infiltration for a given tissue sample. While the model is only supervised using a slide-level class label, it automatically learns to pay attention to the corresponding TIL region. In clinical settings, this model can serve as a pre-selection tool to categorize patients based on high or low TIL infiltration levels. This is particularly useful for clinical trials where TIL levels are of interest.

We compare ANSAC with NIC-CNN, CLAM, and CLAM-MoCo using both quantitative (AUC) and qualitative (visualization of heatmaps) measures. We observed that compared to CLAM, CLAM-MoCo has an advantage through its histologically relevant, MoCo-based feature extractor; and compared to NIC-CNN, CLAM and CLAM-MoCo, ANSAC gains a notable advantage through its segmentation-based attention model. While CLAM-MoCo outperformed CLAM across all datasets, their generated heatmaps showed potential for improvement in accurately identifying relevant regions for the classification task. In contrast, ANSAC’s unique segmentation-based attention model was able to provide more informative heatmaps. In the absence of a pre-trained segmentation model, CLAM-MoCo can achieve good classification performance. However, in the presence of a pre-trained segmentation model, ANSAC can achieve better classification performance as shown by its superior heatmap visualizations.

There are several avenues of interest for future work. Firstly, ANSAC currently employs a straightforward attention mechanism comprising fully connected layers to calculate attention scores. For future investigations, exploring more advanced variations, such as multi-head attention inspired by the popular vision transformer, may help enhance performance. Moving forward, while ANSAC utilizes a two-step approach to compress and process WSIs, future research could explore streamlining this process into a single flow by leveraging advanced architectures such as hierarchical vision transformers^35^. Additionally, if a sufficiently representative dataset becomes available, extending ANSAC to predict multi-class or continuous TIL classifications could be pursued. Furthermore, future research could assess ANSAC’s performance in scoring TILs across individual breast cancer subtypes or even other tumor types, provided datasets for those specific cancer types become accessible. Expanding the ANSAC pipeline to incorporate TIL scoring for survival prediction presents another avenue for broader clinical applications including the ability to assess the method’s ability to enhance the prognostic value of digital TIL analysis. Additionally, refining the segmentation model in ANSAC to handle a mixture of tissue types, such as FFPE and FF, could be explored if more FF tissues become available. Moreover, future endeavors may include further optimization of the MoCo feature extractor used in ANSAC by training it on larger datasets, provided sufficient computation resources are available. Lastly, while incorporating spatial information into TIL analysis offers potential enhancements in prognostic value, it also introduces complexities due to sample biases. Therefore, exploring avenues to standardize digital TIL scores for clinical usage could be a valuable focus of future work. Overall, our promising results establish a foundation for future research directions, particularly in annotation-efficient processing of WSIs, and serve as a starting point for the development of advanced WSI processing tools for tasks where extensive annotations are not readily available.

## Methods

### ANSAC

#### WSI compression using a pre-trained feature extractor

To efficiently process the large-scale WSI with its corresponding slide-level label effectively while minimizing the need for computational resources, we first compressed each gigapixel WSI into a smaller size using the methodology proposed by Tellez et al.^18,19^. In this method, WSI $w$ with dimensions $M\times N\times 3$ (where $M,N$, and 3 represent the image’s height, width, and RGB channel depth) is divided into non-overlapping patches, ${p}_{i,j}$ with dimensions $R\times R\times 3$, where $i=1,\ldots ,\frac{M}{R},j=1,\ldots ,\frac{N}{R}$ and $R$ is the height and width of the patch as shown in Fig. 1a-i^18^. Next, each patch ${p}_{i,j}$ at coordinates $(i,j)$ within the WSI is encoded into a $C$-dimensional vector, ${p}_{{enc}(i,j)}$ using a pre-trained feature extractor ${E}_{\theta }$ (Eq. (1)) as shown in Fig. 1a-ii, a-iii. These encoded vectors are then arranged in a 3-dimensional grid while preserving their original coordinates $(i,j)$, generating the feature-embedding based compressed representation for the WSI, ${w}_{{enc}}\in {{\mathbb{R}}}^{\frac{M}{R}\times \frac{N}{R}\times C}$ (Eq. (2)) as shown in Fig. 1a-iv. For this study, we set $M=N=20480,R=$ 256 and $C$ = 128.

1 $${p}_{i,j}\in {{\mathbb{R}}}^{R\times R\times 3}{\to }^{{E}_{\theta }}{p}_{{enc}(i,j)}\in {{\mathbb{R}}}^{1\times C}$$

2 $$w\in {{\mathbb{R}}}^{M\times N\times 3}{\to }^{{E}_{\theta }}{w}_{{enc}}\in {{\mathbb{R}}}^{\frac{M}{R}\times \frac{N}{R}\times C}$$

To train ${E}_{\theta }$ we utilized self-supervised learning (SSL), which has proven to be advantageous in label and data-scarce domains such as in medical research^18,25,26,36,37^. Specifically, we employed MoCo^25^, which is a SSL-based approach, to train our encoder ${E}_{\theta }$. The MoCo algorithm uses contrastive learning and momentum-based weight updates to learn feature embeddings, giving rise to its name “Momentum Contrast” shortened as “MoCo”. Contrastive learning is an SSL method that uses positive and negative pairs of examples to learn representations that maximize the similarity between the positive pairs and minimize the similarity between the negative pairs. Recent studies have demonstrated that MoCo is a popular pretext task for representation learning in medical image analysis and has outperformed other SSL-based approaches such as SimCLR in medical image classification tasks^38^. Therefore, we choose MoCo as the SSL approach to train ${E}_{\theta }.$

The MoCo-based encoder ${E}_{\theta }$ was trained using a subset of patches (n $\approx {{{\mathrm{210,000}}}}$) extracted from WSIs in the ${D}_{{cleo}}^{{train}}$ dataset and subsequently used as a feature extractor for all datasets. We utilized the Resnet50 architecture as the MoCo backbone, with feature dimension, momentum, Softmax temperature, and queue size set at $128$, ${{{\mathrm{0.999,0.07}}}}$, and $65536$, respectively^25^. The feature extractor was trained for $100$ epochs, and the checkpoint at the epoch with the highest top-1 training accuracy was used to extract a 128- dimensional feature vector for each patch in the WSI.

#### WSI compression guided by pre-trained segmentation network

To automatically segment tissue regions, ANSAC uses an intermediate layer ${S}_{\theta }$ extracted from a convolutional segmentation network trained by Amgad et al.^13^. The segmentation network was made up of a VGG-16, Fully Convolutional Neural Network (FCN-8) architecture and was trained using a dataset consisting of $151$ FFPE WSIs from the TCGA-BRCA Project along with annotations of tissue regions. It was trained to identify four main tissue regions, namely “tumor”, “stroma”, “inflammatory infiltrates”, “necrosis”, and classify the remaining regions as either “other” or “background” regions^13^ (refer Supplementary Fig. 3). To reduce computational complexity and resources, we use the output from the intermediate ${S}_{\theta }$ layer in the segmentation network as the encoding ${p}_{{seg}(i,j)}$ where ${p}_{{seg}(i,j)}\in {{\mathbb{R}}}^{D\times 24x24}$ for a given input patch ${p}_{i,j}$ (Eq. (3)). We also conducted a visual inspection to ensure that this intermediate output contains the same information as the output from the final layer, albeit in a downsampled form. To simplify the dimensions further, we flatten ${p}_{{seg}(i,j)}$ to have $D\times Z$ dimensions where $Z$=576. We obtain such ${p}_{{seg}(i,j)}$ encodings for each patch within the WSI and arrange them following the Tellez et al.^18^ method as in the feature-embedding based compression performed above. This generates a segmentation-based compressed representation of the WSI, ${w}_{{seg}}$ (Eq. (4)) (see Fig. 1a-viii).

3 $${p}_{i,j}\in {{\mathbb{R}}}^{R\times R\times 3}{\to }^{{S}_{\theta }}{p}_{{seg}(i,j)}\in {{\mathbb{R}}}^{D\times Z}$$

4 $$w\in {{\mathbb{R}}}^{M\times N\times 3}{\to }^{{S}_{\theta }}{w}_{{seg}}\in {{\mathbb{R}}}^{\frac{M}{R}\times \frac{N}{R}\times D\times Z}$$

#### ANSAC’s attention-guided learning

While ${w}_{{enc}}$ can be directly processed in a conventional DNN^18^, we drew inspiration from previous work on attention-based approaches for WSI processing^17,39,40^, and developed an attention module, ${A}_{\theta }$. This module was designed as a shallow, wide CNN that outputs attention maps to guide the high-importance regions in each WSI. The attention maps are computed based on the region information from the segmentation map ${w}_{{seg}}$. The attention model consists of $D$ parallel attention branches corresponding to the $D$ regions identified by the segmentation model as shown in Fig. 1b-iii. The individual branches of ${A}_{\theta }$ perform region-specific computations before combining the outputs to produce a final attention score for every patch in the WSI.

To execute the attention computation described above, the segmentation map from Eq. (4) is split into 6 region-specific maps, each with a resolution of ${{\mathbb{R}}}^{\frac{M}{R}\times \frac{N}{R}\times Z\times 1}$ (refer Fig. 1b-iii column 1). These maps are input into the corresponding region-specific branch, where each branch consists of several stacked fully connected layers, ${F}_{1}\in {{\mathbb{R}}}^{512\times 256}$, ${F}_{2}\in {{\mathbb{R}}}^{512\times 256}$ and ${F}_{3}\in {{\mathbb{R}}}^{256\times 1}$ (refer Fig. 1b-iii column 2). The attention for the ${k}^{{th}}$ patch belonging to the ${d}^{{th}}$ region denoted as ${h}_{d,k}$, is computed using the region-specific attention branch, as shown in Eq. (5). The outputs from all region-specific attention branches are summed to obtain the final attention ${a}_{k}$ for the ${k}^{{th}}$ patch as shown in Eq. (6). The attention map for the entire WSI obtained by computing the ${a}_{k}$ value for each patch is referred as ${w}_{{attn}}$ (refer Fig. 1b-iii column 3). ${w}_{{attn}}$ is normalized between 0 and 1, such that each patch that contains a more important region has a high value (closer to 1), and a patch that contains a less important region has a low value (closer to 0). Then it is reshaped to match the height and width of the compressed WSI as shown in Fig. 1b-iv. Finally, the slide-level attention weighted representation for the WSI, denoted as ${w}_{{weighted}}\in {{\mathbb{R}}}^{\frac{M}{R}\times \frac{N}{R}\times 3},$ is obtained through element-wise matrix multiplication of ${w}_{{enc}}$ and ${w}_{{attn}}$ using Eq. (7) (Fig. 1b-v).

5 $${h}_{d,k}=\frac{{e}^{{F}_{3}({relu}({F}_{1}{z}_{k})\, \odot \, {sigm}({F}_{2}{z}_{k}))}}{{\sum }_{j=1}^{K}{e}^{{F}_{3}({relu}({F}_{1}{z}_{k})\, \odot \, {sigm}({F}_{2}{z}_{k}))}}$$

6 $${a}_{k}={\sum }_{d=0}^{D}{h}_{d,k}$$

7 $${w}_{{weighted}}={w}_{{enc}}\odot {w}_{{attn}}$$

The weighted, compressed representation ${w}_{{weighted}}$ is then input into a CNN network $({C}_{\theta })$ as shown in Fig. 1b-vi. The CNN consists of four blocks, each composed of a 2D convolution layer, a Group Normalization layer, and a Rectified linear (ReLU) activation layer. The features obtained from the convolution blocks are then pooled, flattened, and fed into a fully connected layer, which generates the slide-level class prediction, as depicted in Fig. 1b-vii. As mentioned above, ${C}_{\theta }$ model architecture was also used to process the NIC-compressed image in the NIC-CNN baseline method. During training, the binary cross-entropy loss is calculated based on the class prediction of high vs. low TIL infiltration and is used to update the weights in both the attention and classification networks.

#### Model implementation and training

The model parameters are updated via a Stochastic Gradient Descent (SGD) optimizer with a learning rate of $1\times {10}^{-3}$ and a momentum of 0.9. The learning rate was set to decay by a pre-defined value when training reaches 120 and 180 epochs. To improve the model’s generalization and regularization, data augmentation was employed, involving a permutation of common augmentations, such as ${90}^{\circ },{180}^{\circ },{270}^{\circ }$ rotations, and horizontal and vertical flips. A batch size of 16 was used, and the early stop counter was set at 25 to prevent overfitting. A balanced batch sampler was also utilized to address the class imbalance issue, ensuring that each mini-batch had an equal distribution of all classes. The model was trained for ten pre-defined random seeds to ensure reproducibility and robustness. The training was terminated once the validation loss stopped improving beyond 25 epochs (early stop count), and the model with the lowest validation loss was selected as the final checkpoint for the test set. During inference, ANSAC processes and predicts the class label for a WSI within an average of 17 milliseconds. A PyTorch implementation of the ANSAC pipeline, including the segmentation model, will be made publicly available for use.

All evaluated models were trained on 4 Intel(R) Xeon(R) v100, 16GB VRAM GPUs. The pipeline was implemented in Python (version 3.7.4) and Pytorch (version 1.9.0) deep-learning library. It also uses other python supported libraries such as open-slide (version 1.1.2), OpenCV (version 4.2.0.34) and pillow (version 7.2.0), sk-learn, matplotlib (version 3.2.1), NumPy (version 1.17.3), scikit-learn (version 0.23.2), seaborn (version 0.10.1), tensorboard (version 1.15.0), TensorFlow (version 1.15.2), torchsummary (version 1.5.1), torchvision (version 0.10.0), umap-learn (version 0.4.6).

### Comparative analysis using CLAM

CLAM uses concepts from MIL and clustering in its pipeline. It also employs an attention-based approach to assign weights to patches in a WSI before feeding them into the final classification layer. ANSAC is inspired by these ideas in CLAM, such as encoding patches extracted from WSI using feature embeddings and using a multi-branched attention network for attention weight training. However, ANSAC differs from CLAM as it arranges the extracted feature embeddings in their original coordinates and thereby makes use of the patch location information during the classifier training. Additionally, ANSAC introduces multiple attention branches to account for the number of classes obtained from the segmentation map, whereas CLAM adds N parallel attention branches to account for the N classes present in the classification task.

#### Training details

CLAM uses 10-fold cross-validation for model training and evaluation. However, to ensure a fair comparison with ANSAC, we made minor adjustments to the source code to run on ten random weight initializations (seeds) but with the same train, validation, and test divisions across all seeds. All other parameters and training details were kept as in the source code for CLAM. CLAM was trained on 1 Intel(R) Xeon(R) v100, 16GB GPU per RAM GPU.

### Extension on CLAM

We replace CLAM’s feature extractor which was pre-trained on the ImageNet dataset^23^ with the MoCo-based feature extractor, ${E}_{\theta }$ pre-trained on histology images. As mentioned above, ${E}_{\theta }$ converts each $256\times 256$ image into a $128$-dimensional feature vector. The original CLAM model uses 1024, 512 and 256 as the number of neurons in its network layers to accommodate the feature embedding extracted from the ImageNet pre-trained model. For our extension, these were changed to 128, 100, and 64 respectively to account for the feature embedding extracted from ${E}_{\theta }$.

#### Training details

Similar to CLAM, CLAM-MoCo was also run on 10 random weight initializations (seeds) but with the same train, validation, and test division across all seeds to maintain fair comparisons. All other parameters and training details were kept as provided by the authors in their source code for CLAM. CLAM-MoCo was trained on 1 Intel(R) Xeon(R) v100, 16GB GPU per RAM GPU.

### WSI datasets

Supplementary Tables 1 and 2 includes a detailed description of the composition of the individual and mixed datasets. Each WSI dataset contains Breast Cancer (BC) tissue sections annotated by a board-certified breast pathologist with the corresponding TIL score $(0-100 \% )$ following the recommendations of the international working group^5^. Accordingly, 747 Her 2 + BC tissues from the CLEOPATRA clinical study^28^ $({{{{{\rm{D}}}}}}_{{{{{\rm{cleo}}}}}})$, 651 ER + HER2-, Her 2+, TNBC (Triple Negative Breast Cancer) tissues from the FINHER clinical study^29^ (${{{{{\rm{D}}}}}}_{{{{{\rm{fin}}}}}}$), 520 TCGA-BRCA tissues from TCGA (${{{{{\rm{D}}}}}}_{{tcga}}$) and 82 Her2+ and TNBC tissues from the TIGER challenge (${D}_{{{{{\rm{tiger}}}}}}$) along with their TIL scores were made available for model training and evaluation. While ${{{{{\rm{D}}}}}}_{{{{{\rm{cleo}}}}}},{{{{{\rm{andD}}}}}}_{{fin}}$ are in-house datasets, ${{{{{\rm{D}}}}}}_{{tcga}}$ and ${D}_{{{{{\rm{tiger}}}}}}$ WSI datasets are publicly available. For ${D}_{{{{{\rm{tiger}}}}}}$, we only use the TIGER challenge dataset subset for which the TIL scores are provided ($n=82$) (refer https://tiger.grand-challenge.org/Data/ for more details).

While TIL scores are assigned as percentages between 0 and 100% by annotators, we convert the task to a classification task by using a threshold to define two sets of classes as high vs low TIL infiltration. Here, for a given threshold,$t$, all scores below the threshold are labeled as having low TIL infiltration, and all scores above or equal to the threshold are labeled as having high TIL infiltration. Doing so presents us with a binary classification problem instead of the regression problem which is even more challenging to solve using only slide-level labels as ground truth. To ensure that the binary classes are defined in a clinically meaningful manner, it is vital to select an appropriate $t$ value to use as the threshold. In this work, we consider a clinically important factor, i.e., tissue disease stage, to determine the threshold for each dataset. Accordingly, for the datasets containing metastatic tissues (${{{{{\rm{D}}}}}}_{{{{{\rm{cleo}}}}}}$), we set $t$ at $20 \%$ and for the remaining datasets which all contain early-stage tissues, we set $t$ at $30 \%$. Additionally, while the datasets used in our experiments contained a mixture of BC subtypes, including ER + HER2−, HER2+, and TNBC, the analytical validity of models was evaluated independent of the BC subtype across all experiments.

### WSI pre-processing

#### Extracting tissue regions on WSIs

Some WSIs from ${{{{{\rm{D}}}}}}_{{tcga}}$ and ${D}_{{{{{\rm{tiger}}}}}}$ datasets contained two/three tissue sections on a single WSI. Upon verification with our pathologists that a single tissue section is sufficient for model training, we divided the WSI into sub-sections where each section contained only a single tissue image and used one such section as the final WSI. While doing so manually is one option, we used a Python script to automate the process and reduce the manual intervention as much as possible. While the script could split a majority of WSIs correctly, there were a few instances where manual intervention was needed to refine the final output.

#### Tissue segmentation and patching

Given that digitized WSIs contain foreground and background regions, we used a filter on WSIs to filter out non-tissue regions. Each WSI was first resized into a common pixel size and divided into patches of size $256\times 256$ pixels. The resulting $6400$ patches for each WSI were arranged in a $80\times 80({{{{\rm{height}}}}}\times {{{{\rm{width}}}}})$ 2D matrix while preserving the original coordinates. A pre-processing script by IBM CODAIT Centre for Open-source Data & AI Technologies (https://github.com/CODAIT/deep-histopath) was used to filter and retain the foreground patches in each image. Patches detected as background were replaced with a black patch. After filtering, we saved the stack of patches in a NumPy file along with their coordinates.

### Heatmap generation

We adapt the approach used in Lu et al.^17^ to visualize the output from the attention model (attention map) in the form of a heatmap. As done in the previous work, the attention map is converted to percentiles, normalized, and then mapped to the original spatial coordinates to obtain the corresponding heatmap.

### Reporting summary

Further information on research design is available in the Nature Portfolio Reporting Summary linked to this article.

### Supplementary information

Supplementary Information

Reporting Summary

## Data Availability

TCGA slide image data can be accessed through the Genomic Data Commons (GDC) portal https://portal.gdc.cancer.gov/. TIL scores for the TCGA slides are available from the authors upon request. TIGER slide image data, along with TIL scores, are publicly available at https://tiger.grand-challenge.org/. The slide images used in this study from CLEOPATRA and FINHER clinical trials are subject to strict control and usage requirements and have been used with direct permission from the data custodians.

## Code availability

The ANSAC pipeline for pre-processing WSIs, model training, and evaluation can be accessed at https://github.com/rashindrie/ANSAC. The pipeline is provided under the CC BY-NC 4.0 license.
