# Supplementary material for: Annotation-efficient deep learning for breast cancer whole-slide image classification using tumour infiltrating lymphocytes and slide-level labels
Source: Commun Eng. 2024 Jul 25;3:104. doi: 10.1038/s44172-024-00246-9 (PMC11269727; doi:10.1038/s44172-024-00246-9)
Supplement: Supplementary file 1 — Supplementary Information [file 44172_2024_246_MOESM1_ESM.pdf]

Annotation-efficient Deep Learning for Breast Cancer Whole-slide Image Classification using Tumour Infiltrating Lymphocytes and Slide-level Labels

Rashindrie Perera, Peter Savas, Damith Senanayake, Roberto Salgado, Heikki Joensuu, Sandra O'Toole, Jason Li, Sherene Loi, Saman Halgamuge

Supplementary information

WSI Datasets

Supplementary Table 1 describes the four datasets used for training and model validation in this study while Supplementary Table 2 describes the class distribution of each individual and combined dataset.

Supplementary Table 1 I Details including disease stage, TIL threshold, number of low infiltrated WSIs, number of high infiltrated WSI and total number of WSIs in each dataset used in the study.

Dataset	CLEOPATRA (D_{cleo})	FINHER (D_{fin})	TCGA-BRCA (D_{tcga})	TIGER (D_{tiger})
Tissue Type	FFPE	FFPE	Frozen	FFPE
Disease Stage	Metastatic	Early Stage	Early Stage	Early Stage
Threshold	20%	30%	30%	30%
Low Infiltrated WSIs	464	537	520	54
High Infiltrated WSIs	283	114	175	28
Total WSIs	**747**	**651**	**695**	**82**

Supplementary Table 2 I Details of the training, validation, testing set of each dataset used in the study.

Dataset	Class	Training	Validation	Testing
CLEOPATRA (D_{cleo})	Low Infiltration	296	75	93
	High Infiltration	181	45	57
	Total	**477**	**120**	**150**
FINHER (D_{fin})	Low Infiltration	343	86	108
	High Infiltration	73	18	23
	Total	**416**	**104**	**131**
Mix – CF ($\mathrm{D}_{\mathrm{mix\_FFPE}}$)	Low Infiltration	639	161	201
	High Infiltration	254	63	80
	Total	**893**	**224**	**281**

TCGA (D_{tcga})	Low Infiltration	332	84	104
	High Infiltration	112	28	35
	Total	**444**	**112**	**139**
Mix – CFT (D_{mix_all})	Low Infiltration	971	254	305
	High Infiltration	366	91	115
	Total	**1337**	**336**	**420**
TIGER (D_{tiger})	Low Infiltration	-	-	54
	High Infiltration	-	-	28
	Total	-	-	**82**

In both combinations D_{mix_FFPE} and D_{mix_all}, one could argue that we can combine the datasets in a centre-specific manner where a selected set of centres are only in the train set and the rest are in the test set. However, since our goal was to train a generalized model, we choose to first construct the train-validation-test splits for each individual dataset following the 80/20 rule stratified on class label. Each split from the individual centre was then merged to form the corresponding combined mixed split. i.e., the training split from D_{cleo} and D_{fin} were combined to form D_{mix_FFPE} training split which was then combined subsequently with D_{tcga} training split to form D_{mix_all} training split. Constructing our mixed dataset splits in such a manner ensured us that the testing split of D_{mix_all} will be free from training samples in D_{mix_FFPE} thus guaranteeing that the comparison done in Table 1 in the main text is fair. We also believe that such dataset division would ensure that each train, validation, and test set splits have an equivalent and fair distribution of the different types of tissues.

Additional Results

Supplementary Tables 3-7 report the slide-level classification performance for the four models trained on D_i^{train} and evaluated on D_i^{test} while Supplementary Tables 8-12 report the performance for the four models trained on D_i^{train} and evaluated on D_{tiger}^{test}, where $i = cleo, fin, tcga, mix\_FFPE, and\ mix\_all$. Each table reports sensitivity, specificity, balanced accuracy (BA), and Area under the Receiver Operating Curve (AUC) using mean $\pm$ standard deviation across ten random weight initializations.

Supplementary Table 3 I Slide-level classification performance for the four models trained on D_{cleo}^{train} and evaluated on D_{cleo}^{test}.

Model	Sensitivity	Specificity	BA	AUC
NIC-CNN	82.46 $\pm$ 0.040	78.28 $\pm$ 0.031	80.37 $\pm$ 0.016	89.95 $\pm$ 0.013
CLAM	71.93 $\pm$ 0.079	80.00 $\pm$ 0.043	75.96 $\pm$ 0.036	83.51 $\pm$ 0.023
CLAM- MoCo	82.63 $\pm$ 0.038	80.86 $\pm$ 0.033	81.75 $\pm$ 0.025	90.05 $\pm$ 0.012
ANSAC	85.26 $\pm$ 0.029	83.33 $\pm$ 0.027	84.30 $\pm$ 0.013	91.84 $\pm$ 0.007

Supplementary Table 4 I Slide-level classification performance for the four models trained on D_{fin}^{train} and evaluated on D_{fin}^{test}.

Model	Sensitivity	Specificity	BA	AUC
NIC-CNN	80.87 ± 0.042	92.04 ± 0.022	86.45 ± 0.023	90.72 ± 0.022
CLAM	66.52 ± 0.126	92.31 ± 0.015	79.42 ± 0.062	93.46 ± 0.018
CLAM- MoCo	76.52 ± 0.065	94.17 ± 0.017	85.34 ± 0.028	95.77 ± 0.010
ANSAC	85.65 ± 0.071	90.74 ± 0.024	88.20 ± 0.032	95.29 ± 0.015

Supplementary Table 5 I Slide-level classification performance for the four models trained on D_{tcga}^{train} and evaluated on D_{tcga}^{test}.

Model	Sensitivity	Specificity	BA	AUC
NIC-CNN	64.86 ± 0.086	87.88 ± 0.025	76.37 ± 0.035	86.92 ± 0.015
CLAM	30.57 ± 0.255	92.69 ± 0.068	61.63 ± 0.096	79.27 ± 0.078
CLAM- MoCo	70.57 ± 0.041	88.75 ± 0.017	79.66 ± 0.017	87.87 ± 0.015
ANSAC	58.00 ± 0.238	87.88 ± 0.066	72.94 ± 0.095	85.74 ± 0.025

Supplementary Table 6 I Slide-level classification performance for the four models trained on $D_{mix\_FFPE}^{train}$ and evaluated on $D_{mix\_FFPE}^{test}$.

Model	Sensitivity	Specificity	BA	AUC
NIC-CNN	$73.00\% \pm -0.048$	$84.13\% \pm 0.026$	$78.56\% \pm 0.019$	$87.74\% \pm 0.011$
CLAM	$77.88\% \pm 0.067$	$84.13\% \pm 0.032$	$81.00\% \pm 0.024$	$88.65\% \pm 0.008$
CLAM- MoCo	$76.25\% \pm 0.04$	$83.63\% \pm 0.024$	$79.94\% \pm 0.016$	$88.96\% \pm 0.008$
ANSAC	$78.12\% \pm 0.090$	$84.83\% \pm 0.022$	$81.48\% \pm 0.037$	$89.91\% \pm 0.017$

Supplementary Table 7 I Slide-level classification performance for the four models trained on $D_{mix\_all}^{train}$ and evaluated on $D_{mix\_all}^{test}$.

Model	Sensitivity	Specificity	BA	AUC
NIC-CNN	$73.30\% \pm 0.054$	$82.49\% \pm 0.035$	$77.90\% \pm 0.016$	$87.45\% \pm 0.010$
CLAM	$76.17\% \pm 0.046$	$84.89\% \pm 0.034$	$80.53\% \pm 0.014$	$87.16\% \pm 0.009$
CLAM- MoCo	$71.39\% \pm 0.042$	$85.8\% \pm 0.012$	$78.6\% \pm 0.017$	$88.35\% \pm 0.008$
ANSAC	$76.70\% \pm 0.041$	$84.16\% \pm 0.027$	$80.43\% \pm 0.011$	$89.24\% \pm 0.008$

Supplementary Table 8 I Slide-level classification performance for the four models trained on D_{cleo}^{train} and evaluated on D_{tiger}^{test}.

Model	Sensitivity	Specificity	BA	AUC
NIC-CNN	44.64 ± 0.0682	90.19 ± 0.0406	67.41 ± 0.0283	$77.77 \pm .0249$
CLAM	58.21 ± 0.0918	73.15 ± 0.0448	65.68 ± 0.0493	71.38 ± 0.0343
CLAM- MoCo	53.21 ± 0.0806	87.41 ± 0.0369	70.31 ± 0.0273	78.97 ± 0.0187
ANSAC	47.50 $\pm$ 0.0750	90.93 $\pm$ 0.0375	69.21 $\pm$ 0229	80.06 $\pm$ 0.0352

Supplementary Table 9 I Slide-level classification performance for the four models trained on D_{fin}^{train} and evaluated on D_{tiger}^{test}.

Model	Sensitivity	Specificity	BA	AUC
NIC-CNN	08.57 ± 0.0237	100 ± 0.0	54.29 ± 0.0119	56.92 ± 0.0590
CLAM	05.71 ± 0.0510	100 ± 0.0	52.86 ± 0.0255	73.98 ± 0.0486
CLAM- MoCo	21.07 ± 0.1679	95.56 ± 0.0463	58.31 ± 0.0640	82.54 ± 0.0316
ANSAC	12.50 ± 0.0559	100 ± 0.0	56.25 ± 0.0280	66.45 ± 0.0945

Supplementary Table 10 I Slide-level classification performance for the four models trained on D_{tcga}^{train} and evaluated on D_{tiger}^{test}.

Model	Sensitivity	Specificity	BA	AUC
NIC-CNN	43.21 ± 0.1838	75.93 ± 0.1086	59.57 ± 0.0480	66.05 ± 0.0391
CLAM	00.71 ± 0.0214	99.81 ± 0.0056	50.26 ± 0.0079	62.03 ± 0.0636
CLAM-MoCo	35.36 ± 0.2451	80.19 ± 0.1391	57.77 ± 0.0577	66.03 ± 0.0407
ANSAC	34.64 ± 0.1924	81.48 ± 0.1165	58.06 ± 0.0420	65.76 ± 0.0468

Supplementary Table 11 I Slide-level classification performance for the four models trained on $D_{mix\_FFPE}^{train}$ and evaluated on D_{tiger}^{test}.

Model	Sensitivity	Specificity	BA	AUC
NIC-CNN	37.86 $\pm$ 0.1219	96.11 $\pm$ 0.0365	66.98 $\pm$ 0.0467	81.92 $\pm$ 0.0261
CLAM	56.43 $\pm$ 0.06	82.22 $\pm$ 0.046	69.33 $\pm$ 0.018	76.82 $\pm$ 0.021
CLAM- MoCo	73.21 $\pm$ 0.045	74.81 $\pm$ 0.043	74.01$\pm$ 0.014	81.71 $\pm$ 0.016
ANSAC	46.07 $\pm$ 0.1648	91.30 $\pm$ 0.0636	68.68 $\pm$ 0.0546	85.13 $\pm$ 0.0203

Supplementary Table 12 I Slide-level classification performance for the four models trained on $D_{mix\_all}^{train}$ and evaluated on D_{tiger}^{test}.

Model	Sensitivity	Specificity	BA	AUC

NIC-CNN	59.64 ± 0.1217	82.96 ± 0.0794	71.30 ± 0.0315	81.87 ± 0.0260
CLAM	35.71 ± 0.058	92.04 ± 0.042	63.88 ± 0.032	75.44 ± 0.037
CLAM- MoCo	61.07 ± 0.072	83.7 ± 0.078	72.39 ± 0.022	80.98 ± 0.021
ANSAC	60.71 ± 0.1307	83.33 ± 0.0764	72.02 ± 0.0416	83.40 ± 0.0223

Visualization and interpretability of model predictions.

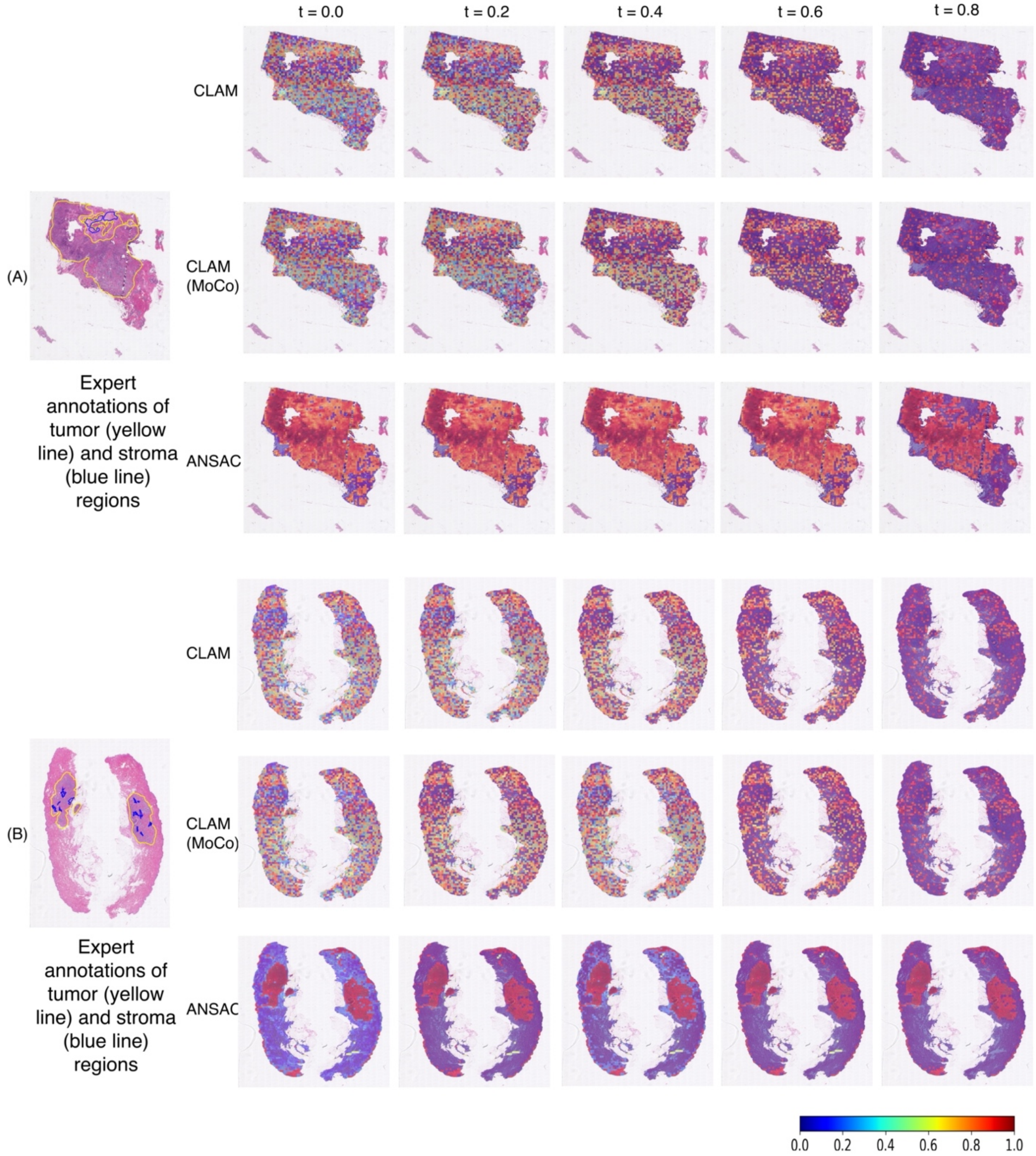

Supplementary Figure 1: $\mathbf{A}, \mathbf{B}$: Two example slides from $\mathbf{D}_{\mathbf{cleo}}^{\mathbf{test}}$ with Low TIL infiltration. Two example slides with **low TIL infiltration** are presented in panels A and B. While Figure 2 in the main text illustrated the model predictions for only four different threshold settings, t (where $t = 0,\ 0.4, 0.6\ and\ 0.8$) due to space limitations, here we also present the predictions for $t = 0.2$.

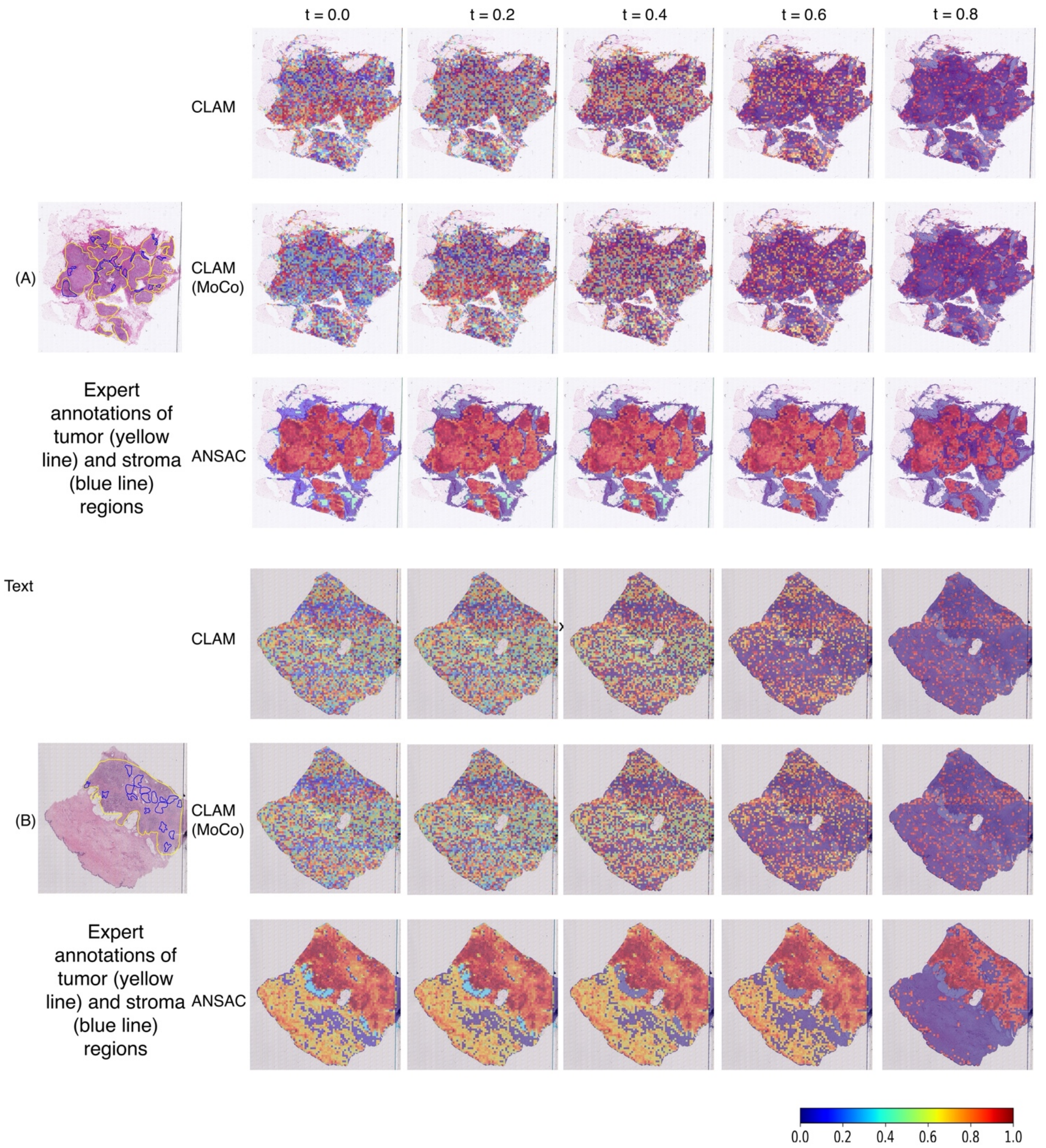

Supplementary Figure 2: $\mathbf{A}, \mathbf{B}$: Two example slides from $\mathbf{D}_{\mathbf{cleo}}^{\mathbf{test}}$ with High TIL infiltration. Two example slides with **High TIL infiltration** are presented in panels A and B. While Figure 3 in the main text illustrated the model predictions for only four different threshold settings, t (where $t = 0,\ 0.4, 0.6\ and\ 0.8$) due to space limitations, here we also present the predictions for $t = 0.2$.

Example segmentation maps for WSIs in $\mathbf{D}_{\mathbf{cleo}}$ dataset

Below we share the segmentation maps obtained from the pre-trained segmentation model[16] used in ANSAC for the four WSIs shown in Fig. 2 and 3 in the main text. We note how the borrowed segmentation labelling led to noisy labels in several regions. This is expected given that the segmentation model was neither trained

nor fine-tuned on any of the datasets used in this work. However, although noisy, the usefulness of the segmentation labels for ANSAC on learning the classification task was evident when comparing the reported results as well as the visualisation of the attention heatmaps generated from ANSAC.

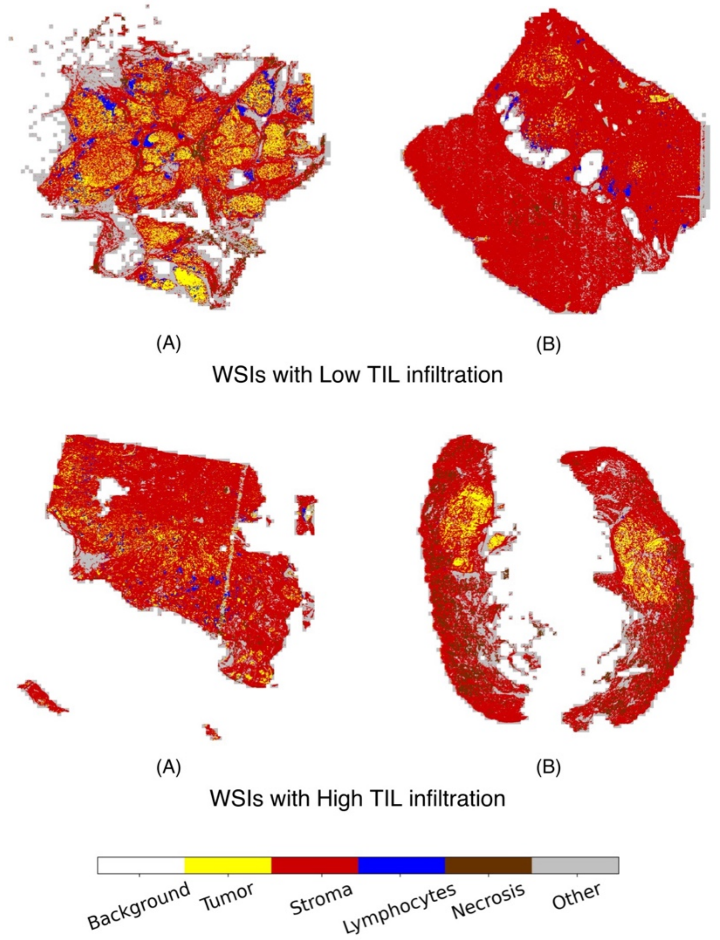

Supplementary Figure 3: Visualization of the pre-trained, borrowed segmentation predictions used as inputs in ANSAC. Top: A-B: Visualise the two WSIs used in main text Fig. 2 while **bottom: A-B:** visualise the two WSIs used in main text Fig. 3. The colour bar denotes the colour labels used for the five tissue regions (tumour, stroma, lymphocytes, necrosis and other) identified by the segmentation model with the background region marked in white.
